# How snoRNAs can contribute to cancer at multiple levels

**DOI:** 10.1093/narcan/zcae005

**Published:** 2024-02-24

**Authors:** Federico Zacchini, Chiara Barozzi, Giulia Venturi, Lorenzo Montanaro

**Affiliations:** Departmental Program in Laboratory Medicine, IRCCS Azienda Ospedaliero-Universitaria di Bologna, Via Albertoni 15, I-40138 Bologna, Italy; Department of Medical and Surgical Sciences (DIMEC), Alma Mater Studiorum - University of Bologna, Bologna I-40138, Italy; Department of Medical and Surgical Sciences (DIMEC), Alma Mater Studiorum - University of Bologna, Bologna I-40138, Italy; Centre for Applied Biomedical Research – CRBA, University of Bologna, Sant’Orsola Hospital, Bologna I-40138, Italy; Departmental Program in Laboratory Medicine, IRCCS Azienda Ospedaliero-Universitaria di Bologna, Via Albertoni 15, I-40138 Bologna, Italy; Department of Medical and Surgical Sciences (DIMEC), Alma Mater Studiorum - University of Bologna, Bologna I-40138, Italy

## Abstract

snoRNAs are a class of non-coding RNAs known to guide site specifically RNA modifications such as 2′-O-methylation and pseudouridylation. Recent results regarding snoRNA alterations in cancer has been made available and suggest their potential evaluation as diagnostic and prognostic biomarkers. A large part of these data, however, was not consistently confirmed and failed to provide mechanistic insights on the contribution of altered snoRNA expression to the neoplastic process. Here, we aim to critically review the available literature on snoRNA in cancer focusing on the studies elucidating the functional consequences of their deregulation. Beyond the canonical guide function in RNA processing and modification we also considered additional roles in which snoRNA, in various forms and through different modalities, are involved and that have been recently reported.

## Graphical Abstract

**Graphical Abstract:**
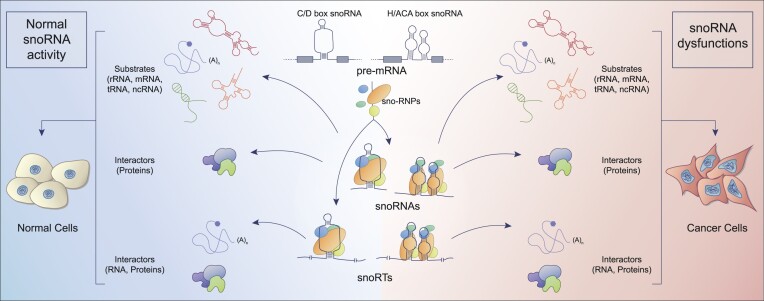

Small nucleolar RNAs (snoRNAs) are a class of non-coding RNAs that, soon after their identification, attracted a growing interest at different levels. On one hand the information available on their biogenesis, molecular features, protein interactors and functions, grew rapidly (1–4). In fact, although some aspects are still under definition, a considerable degree of consensus has been reached in this sense. On the other hand, while it appeared clear very early that snoRNA are strongly deregulated in some human disorders and particularly in cancer, a comprehensive understanding of their contribution to the molecular pathogenesis of these conditions is still lacking. Currently, snoRNAs are often proposed as potential biomarkers in different malignancies (see (5–7) for review) but one major obstacle in their recognition in this sense is the general lack of insight concerning the biological significance of their alteration. In the present review we aim to connect the available information regarding the changes of snoRNAs reported in cancer with their functional consequences. In our view, this will provide the reader with a key to understanding the role of this class of RNAs in cancer. For this purpose, a concise summary reporting snoRNAs features and functions will be first provided.

## Features and functions of snoRNA

**Figure 1.**
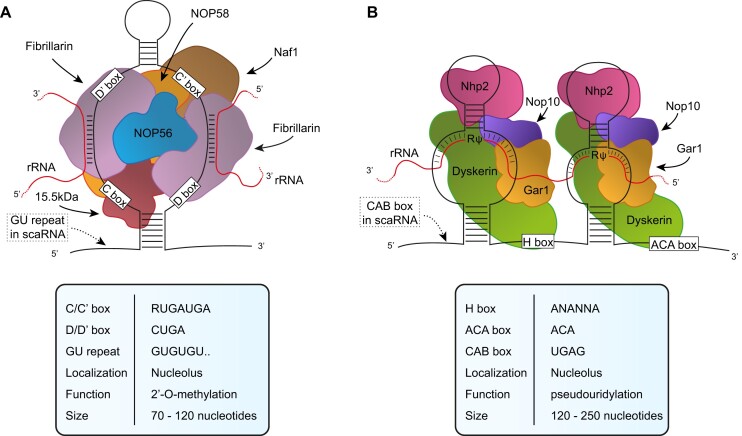
snoRNP structures. Simplified representation of **(A)** C/D box snoRNP and **(B)** H/ACA box snoRNP. The distinctive features are detailed in the tables below. GU repeat and CAB box are represented as dotted box and are related to C/D and H/ACA scaRNAs, respectively.

snoRNAs are a class of small RNAs with a characteristic secondary structure initially identified in the nucleolus of the cell where they carry out their most known functions: process and modify nascent ribosomal RNA (rRNA). They have been classified in two major groups: C/D box and H/ACA box snoRNAs. C/D box snoRNAs are characterized by two sequence motives: box C and Box D present in pairs in a single molecule (Box C/C′ and box D/D′). Due to sequence complementarity, they are folded in a hairpin secondary structure with a larger central loop that places in near proximity C/C′ and D/D′ motives (8,9). C/D Box snoRNAs assemble with four core proteins to form small nucleolar ribonucleoprotein (snoRNPs) in which fibrillarin (FBL) exerts the catalytic activity which consists in methylation of the 2′ hydroxyl group of ribose in RNA. The second class of snoRNA is represented by H/ACA Box snoRNAs. They are characterized by the presence of two motives: a central motive termed box H and a terminal one, the Box ACA that remain single strand while the rest of the molecule folds in two stem-loops separated by the H Box. Each hairpin allows the binding of four core proteins including dyskerin which carry the catalytic pseudouridylation activity. In addition to the two major categories described so far, there is a minor set of snoRNAs that has been identified within Cajal bodies and therefore named small Cajal bodies RNAs (scaRNAs). They are characterized by the presence of the Cajal body localization signal (CAB box) and either C/D box or H/ACA box or both sequence motives (10,11). The main differences between the two groups of snoRNAs are their localization and RNA substrate: canonical C/D box and H/ACA box are mainly localized in the nucleolus and their main substrate is ribosomal RNA, while scaRNA localize in Cajal bodies and modify other small nuclear RNAs. The main structural features are outlined in Figure 1.

In humans the majority of snoRNA sequences are positioned in introns of highly transcribed genes involved in ribosome biogenesis and in translation, except a few of them that are transcribed independently (12). Indeed, more than one snoRNA sequence can be hosted in one single host gene, e.g. RPL7A or eIF4A2 in which several snoRNAs can be found. There are also non-coding genes that include many snoRNAs in their sequence, for example UHG, GAS5, U17HG, U19HG and U50HG. Remarkably, all snoRNA host genes present 5′ terminal oligopyrimidine (5′TOP) motifs that are reported to regulate translation through a mechanism involving mTOR (13), linking snoRNA host genes to other genes involved in translation. In principle, this system would allow a balanced expression of snoRNAs together with all the factors involved in ribosome biogenesis and translation, such as ribosomal proteins or translation initiation factors. However, it has been reported that a great number of transcripts resulting from snoRNAs alternative splicing are not translated into proteins but are directed to degradation via nonsense-mediated RNA decay (NMD) (14). Therefore, this mechanism suggests that snoRNAs expression can be regulated independently of the expression of the transcript deriving from the same host gene (15). Since snoRNAs are included in pre-mRNA transcripts, they are transcribed by RNA Pol II and extensively processed to reach mature conformation (15) via two main pathways: splicing-dependent pathway and splicing-independent pathway (16). Afterwards, they are assembled with the core proteins to form active small nucleolar ribonucleoprotein particles (snoRNPs) and transported in the nucleolus (17), where they carry out their main function.

Although in recent years different functions of snoRNAs has emerged, describing how they may contribute to the biology of the cell and linking their expression to the behavior of cancer cells, the first described function of snoRNAs to be reported was their involvement in rRNA processing. In fact, SNORD3 snoRNA (U3) was initially reported to mediate the assembly of the small subunit processome (SSU), a complex responsible for the maturation of 18S rRNA and the 40S subunit (18). It has been reported that U3 interacts through base pairing with the external transcribed spacer (ETS) positioned at the 5′ of the 47S rRNA precursor (19) guiding the initial cleavage that starts rRNA processing. More recently, several other C/D Box snoRNAs guiding rRNA processing have been described, for instance SNORD118 (U8) and SNORD13 (U13) (1) and SNORD14 (U14) for which is not clear if the action on rRNA processing is due to its activity of 2′-O-methylation (20). In addition, other snoRNAs have been reported to be involved in rRNA processing, i.e. SNORD22 (U22) (21), SNORA73 (U17) (22), SNORA62 (E3) and SNORA63 (E3) (23) but their mechanism of action is still uncharacterized.

During ribosome biogenesis rRNA is processed but also extensively modified and, as mentioned before, snoRNAs and snoRNPs are mainly responsible for these modifications. C/D Box snoRNPs containing fibrillarin catalyze ribose 2′-O-methylation co-transcriptionally guided by sequence complementarity of specific snoRNAs and using S-adenosyl methionine (SAM) as the methyl donor. Methylation of RNA increases resistance to hydrolysis and reduces the flexibility of the molecule by restricting the 3′-phosphate rotational freedom (24,25). This contributes to rRNA stabilization and functionality, allowing interactions with RNA and proteins in important regions of the ribosome (26). Recent RiboMeth-seq results demonstrated that one third of the known sites have a variable level of modification in human cell lines (27–30) suggesting that human cells tolerate ribosomes with a reduced level of methylation. These results paved the way for methylation to be recognized as a source of ribosome heterogeneity (31). At this regard, a very interesting study pointed out that stable fully methylated sites are positioned in the core of the ribosome on important functional sites, whereas the more variable sites are located at the periphery of the ribosome (32). Specifically, the modulation of 2′-O-methylation profile seems to impact on the translation of a subset of mRNAs, such as the ones containing an internal ribosome entry site (IRES) element (28) suggesting the importance of this modification in translation initiation. On the other hand, H/ACA Box snoRNPs are responsible for the isomerization of uridine in pseudouridine (Ψ) due to the activity of dyskerin. Like 2′-*O*-methylation, also pseudouridylation occurs during rRNA transcription by direct pairing with specific H/ACA Box snoRNAs for each site. Due to the presence of one more hydrogen bond donor compared to uridine, pseudouridine confers increased rigidity to the phosphodiester RNA backbone, increases the thermal stability of the molecule and, by stabilizing Ψ-A pairing, it plays an important role in the formation of RNA secondary structures (33). In fact, in ribosomes they are able to maintain ribosome structural integrity during translation. Moreover, it has been widely reported that pseudouridylation sites are positioned in important functional areas of the ribosome, such as the decoding site, mRNA channel, peptidyl transferase center, tRNA binding site, and ribosomal subunit interface (34). Therefore, the loss of pseudouridine clusters can alter ribosome assembly and ultimately its translational efficiency by reducing, for example, amino acid incorporation rate or ribosome fidelity (35–37). Since the growing importance that snoRNAs are gaining in cell biology and in cancer, databases that summarize name, sequence and target of each snoRNA were needed. Two of the most complete are snoDB released by the Scott group (https://bioinfo-scottgroup.med.usherbrooke.ca/snoDB/ (38)) and RNA central released by the European Bioinformatics Institute (https://rnacentral.org/ (39)). For a certain number of snoRNA reported in these databases there are no target sequence described yet, therefore, these snoRNAs are known as orphan snoRNAs.

Since the first studies on RNA modification, it was clear that not only rRNA was extensively modified, but also transfer RNAs (tRNAs) and small nuclear RNAs (snRNAs) (40). More recently, new studies suggested that also mRNA, long non-coding RNAs (lncRNAs) and snoRNAs themselves are modified by C/D Box and H/ACA Box snoRNPs guided on the target nucleotide by snoRNAs through sequence complementarity. The modifications may have a similar impact on these RNAs, affecting RNA stabilization and integrity (41).

Other functions of snoRNAs based on RNA-RNA interaction have been described. It has been reported that particular snoRNAs can guide acetylation in rRNA by associating with the acetyltransferase NAT10 (42). Several other snoRNAs are involved in alternative splicing by binding to the pre-mRNA target and promoting the inclusion or the exclusion of an alternative exon (43–46). In particular, SNORD2 was reported to bind to its own host gene transcript and inhibit the inclusion of the downstream alternative exon (47) suggesting a regulatory mechanism. In addition, snoRNAs contribute also to regulate 3′-processing of mRNA due to the interaction with the cleavage and polyadenylation specificity factor (CPSF) (48,49). Finally, a small number of snoRNAs are reported to bind to chromatin and contribute to its remodeling and therefore to gene expression (50–52).

In last years, evidence was provided indicating that snoRNAs can be further processed to originate smaller RNAs named snoRNA derived RNAs (sdRNAs) and piwi interacting RNAs (piRNAs). The first group seems to have miRNA-like biogenesis and functions (53), while the second group is involved in the degradation of transposon RNA and in the epigenetic regulation of gene expression (54). In particular, recent studies suggested that sdRNAs could be involved in human cancer (reviewed in (55)) even though a full characterization of their role in cancer biology is still missing.

## SnoRNA in cancer

For more than two decades, increasing evidence had been gathered on snoRNAs contribution in cancer development. In fact, numerous studies showed the correlation between alterations in the expression of some snoRNAs and different cancer features, like tumor cell proliferation, migration, invasiveness, and its association to patients’ prognosis (56–59). Because of these features, snoRNAs are often suggested as potential biomarkers useful in the assessment of cancer diagnosis/prognosis. This type of evidence has been extensively discussed in recent reviews (60,61) which have highlighted a wide variety of snoRNAs that are dysregulated in many different tumors. This array of identified snoRNAs consistently differs across different types of tumors (and in different studies), which makes it challenging to clearly define their roles as potential diagnostic biomarkers and modulators in cancer progression or tumorigenesis. Additionally, the mechanisms through which they operate in relation to cancer are often poorly defined. However, it is evident that there are increasing efforts in this direction, but few considerations on how to interpret these results are needed. It is important to note that many of the analyses conducted were not optimal for the identification of snoRNAs due to their molecular and structural characteristics, which make their quantification not always reliable in several key points. For example, the extraction of snoRNAs can vary as standard extraction methods do not allow optimal purification of RNAs smaller than 200 nucleotides. Therefore, it is recommended to use more suitable extraction methods ensuring high efficiency for the recovery of both small and large sized RNAs. Another critical step in their analysis is the conversion to cDNA as snoRNAs are particularly difficult to reverse transcribe under standard conditions, due to their highly stable secondary structure, thus introducing a quantification bias. Recent publications suggest the use of reverse transcriptases active up to 60°C, allowing the relaxion of the secondary structure of RNA and therefore facilitating reverse transcription (62). For the same reasons of size and structure it is also difficult in some cases to carry out an appropriate design of the primers or probes for their analysis.

### Alterations in snoRNA-mediated guide functions in cancerous ribosome biogenesis

To understand the role of snoRNAs alterations in cancer biology it is important to evaluate their impact on rRNA processing and modifications. For a detailed overview of the mechanisms involved in ribosome biogenesis, we suggest referring to recent reviews on the topic (63,64) as this falls out of the scope of the present review. Ribosomes are large macromolecular machines composed, in humans, of 80 different structural proteins and 4 rRNAs assembled in a small and large subunit. Ribosome biogenesis is a highly regulated mechanism involving multiple steps leading to nucleolytic cleavage of the rRNA precursor molecules together with the different chemical modifications of specific nucleotides and correct folding of mature subunits. During processing, pre-rRNAs associate not only with ribosomal proteins further retained within mature ribosomes, but also with non-ribosomal proteins and snoRNPs which participate in distinct aspects of this process. As described above, pre-rRNA processing, which occurs early in ribosome biogenesis, is mediated by base-pairing interactions between a limited number of snoRNAs with pre-rRNA (2,65,66) leading to pre-rRNA cleavage and folding (67–69) rather than rRNA modification. Of note, at the beginning of the process, the hybridization of a snoRNA to a given rRNA stretch most likely prevents the misfolding of the respective region. To support cell growth and proliferation, cancer cells are able to modulate the expression of all the components for ribosomal biogenesis to sustain ribosome demand and hyperactive protein synthesis. Also, dysregulation of this subgroup of snoRNAs was linked to the acquisition of certain tumor features such as chemoresistance. For instance, in acute monocytic leukemia (AML) cells, U3 and U8 overexpression due to increased levels of the fragile X mental retardation–related protein 1 (FXR1), a protein associated with ribosomes, was found to be related to higher ribosome gene transcription and 45S rRNA processing (70). Moreover, FXR1 can regulate other C/D box snoRNAs and H/ACA box snoRNAs guiding rRNA modifications resulting in increased level of modification at important sites. This could definitely affect translation enabling the cells to adapt to chemotherapy and survive.

Ribosomal RNA modifications occur co-transcriptionally and are known to play a crucial role in ribosomal activity, mRNA translation, and ultimately gene expression control. Indeed, a set of recent observations suggest the presence of ribosomal subpopulations that exhibit variations in the RNA or protein components (also known as ribosomal heterogeneity and specialized ribosomes) that can modulate the translational program (71,72). This additional level of control of gene expression may be important in the translational imbalance necessary for tumor growth and development. It is well established that a general alteration of rRNA modification can contribute to tumor progression. This was first demonstrated experimentally by altering the expression of specific components (i.e. fibrillarin or dyskerin) of the ribonucleoprotein modification complexes leading to changes in the expression of key cancer genes including p53, p27, XIAP, Bcl-xL (28,73–75). Recently, some additional efforts have been made to dissect the direct function of snoRNAs on specific rRNA modifications to recognize their mechanistic connections on cancer features. Accordingly, a recent study assessed the role of SNORA24 in hepatocellular carcinoma and its loss in tumor initiation and maintenance of RAS-driven cancer (76). SNORA24 guides the conversion of uridine to pseudouridine at positions 609 and 863 on 18S rRNA. Ribosomes with decreased level of these modifications exhibit reduced aminoacyl-tRNA selectivity and fidelity during translation elongation, leading to changes in the abundance of specific proteins which favor cancer cell survival. By contrast, an upregulated expression of a group of H/ACA snoRNAs was identified as a hallmark of high-grade serous ovarian cancer (77). In this study, a dedicated approach suitable for highly structured small to mid-size non-coding RNAs was applied, and it was found that SNORA81, SNORA19 and SNORA56 represented a distinctive H/ACA snoRNA signature that can differentiate between serous high-grade ovarian carcinoma from serous borderline tumor. Specifically, the expression of SNORA81 was linked to rRNA modification levels. The knockdown of SNORA81 inhibited the 4606–28S rRNA pseudouridylation and ribosome biogenesis, resulting in decreased cell proliferation and migration. This further supports the idea that certain subsets of H/ACA snoRNAs may contribute to tumor aggressiveness by influencing rRNA modification and synthesis.

**Figure 2.**
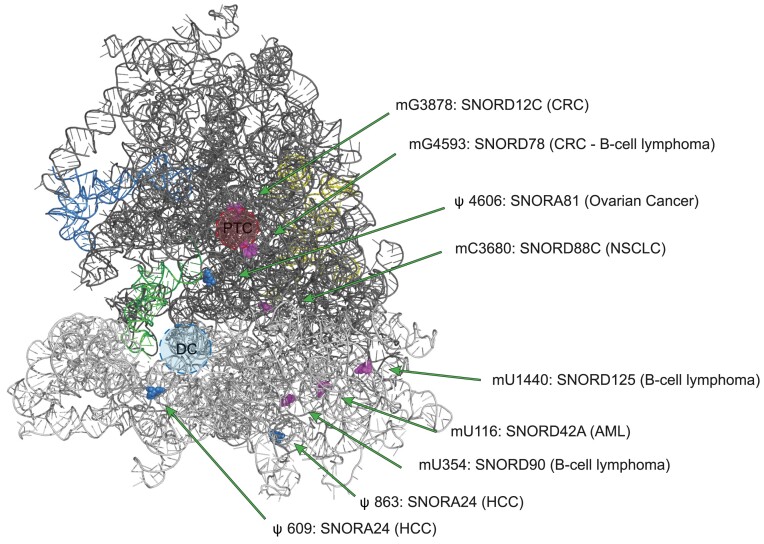
Localization of the modification sites in the 80S ribosome structure targeted by snoRNAs dysregulated in cancer. The structure of 80S ribosome (PDB entry 6QZP) shows only RNAs: 18S (light grey), 28S (dark grey), 5.8S (yellow), 5S (blue) and tRNA (green). Pseudouridine positions modified by the relevant H/ACA box snoRNAs are depicted as blue spheres, while 2′-*O*-methylation sites modified by C/D box snoRNA are depicted as pink spheres. PTC: peptidyltransferase centre; DC: decoding centre; CRC: colorectal cancer; NSCLC: non-small cell lung cancer; AML: acute myeloid leukemia; HCC: hepatocellular carcinoma.

C/D box snoRNAs may also be implicated in promoting or modulating tumor features. As such, SNORD88C was found to play an oncogenic role in non-small cell lung cancer (NSCLC) promoting proliferation and metastasis *in vitro* and *in vivo* (78). SNORD88C acts guiding the methylation of C3680 on 28S rRNA. In NSCLC the overexpression of SNORD88C leads to an increase in the fraction of ribosomes bearing C3680 2′-*O*-methylation associated to a consistent selective increase in the translation of the mRNA encoding for Stearoyl-CoA desaturase 1 (SCD1), a central lipogenic enzyme for a monounsaturated fatty acid synthesis. SCD1 overexpression inhibited lipid peroxidation, affected mTOR pathway and eventually inhibited autophagy, largely considered as tumor suppressive mechanism through cell cycle arrest and maintenance of genome and organelle integrity. This specific feature was directly related to migration and invasion of lung cancer cells. Similarly, analyzing the snoRNA expression patterns in AML patients, SNORD42A was found to be the most highly expressed snoRNA and its role in leukemogenesis was investigated (79). SNORD42A guides 18S-U116 2′-*O*-methylation, which was shown to enhance IRES-dependent translation, mainly increasing the expression of several ribosomal proteins. The authors suggested that this modification, although not so close to the decoding center and the mRNA entry site, may induce a conformational switch of the ribosome which changes the preference for translation initiation, giving a survival advantage to neoplastic cells. In another study it was observed that the lncRNA ZFAS1 (host gene of three members of the SNORD12 family) interacts directly with NOP58, accelerating the assembly of SNORD12C and SNORD78 on the snoRNP complex. This event favors the modification of G3878 and G4593 on the 28S rRNA, guided by SNORD12C and SNORD78 respectively. The alteration on these modifications impacts on the stability and translation of downstream targets of ZFAS1 (such as EIF4A3 and LAMC2), which directly impacts the development of colorectal carcinoma (CRC) (80). Figure 2 shows the localization of the above reported modification sites in the 80S ribosome structure.

In addition to the several studies investigating the effect of the dysregulation of an individual rRNA modification site promoted by a single or few snoRNAs, more recently, the epitranscriptomic rRNA profile was analyzed in cellular models and some tumor types to dissect the translation machinery and possibly find alternative novel potential therapeutic targets for dysregulated ribosomes. Since ribose 2′-*O*-methylation detection has been so far more manageable than pseudouridylation, first reports exploited RiboMeth-Seq, a high-throughput sequencing for RNA ribose methylation profiling, to map and quantify 2′-*O*-methylation rRNA in human tumors. Indeed, a wide intra- and intertumoral heterogeneity in 2′-O-methylation among the 106 sites was observed in large B-cell lymphoma, AML and breast carcinoma, where different methylation profiles were associated with histological features and patients' survival (27,32,81). Regarding large B-cell lymphoma, a comparative analysis of rRNA modification and snoRNA expression was also investigated. The authors demonstrated the hypomodification in a subset of 2′-O-methylation rRNA sites both in cell lines and primary tumor samples. This differed to the full, or nearly full, methylation level of the majority of the positions in several adult tissues or reactive lymph nodes. Furthermore, together with rRNA profiling, RiboMeth-seq provided low-coverage RNA-seq of the snoRNAs that could be representative of their expression levels. There was a remarkable absence of a global correlation between the expression levels of individual snoRNA and the corresponding methylation levels at their respective target sites. The only exception was represented by three of the most dysregulated sites, with the expression levels of guide snoRNAs varying in parallel with the methylation stoichiometry (SNORD90 for 18S-U354, SNORD125 for 18S-C1440 and SNORD78 for 28S-G4593).

Although pseudouridine has been known for >50 years and is considered as the first modified nucleoside that has been identified, the study of the pseudouridylation pattern has been hampered by the limited technical approaches for its detection, due to undistinguishable features and no mass changes of the isomeric form of uridine. Nevertheless, over the last few years, different approaches were developed for the quantitative and site-specific identification of pseudouridine profile in a specific class of RNAs or in the whole transcriptome (82–87). These studies paved the way for a deeper comprehension of the landscape of epitranscriptomics in cellular models and human tumors. Very recently, an extensive dysregulation of the pseudouridylation pattern of rRNA was shown in breast carcinomas (88). In particular, the most dysregulated positions in tumor samples were found to be hypermodified with respect to a reference RNA and alterations of pseudouridylation level at specific rRNA sites could distinguish patients’ clusters with different clinicopathological features. In this study the presence of alterations in the expression of H/ACA box snoRNA guiding the pseudouridine modification in the same tumor was also analyzed. Remarkably, no correlation between snoRNA expression and the modification level was detected, probably due to the limited series size. Although the two reported studies could only prove a certain degree of alteration of snoRNAs expression without analyzing the causes leading to the dysregulation or the biomolecular effects, it is more and more evident that snoRNAs as well as the modification they introduce should be considered as novel layer for ribosome diversity which may in turn influence cancer biology. These studies, exploiting high-throughput methodologies, have highlighted alterations in the profile of major rRNA modifications but have not thoroughly investigated the correlation with snoRNA alterations. Instead, by exploiting the TGIRT-Seq approach (89), it will likely be possible to gain new insights regarding the omic data of snoRNAs and variations in the modification profile of their target RNAs.

The accumulating evidence of the heterogeneous composition of ribosomes led to consider them not merely passive executors of protein synthesis but instead as dynamic players of translation with regulatory function on gene expression. Ribosome heterogeneity could arise from any of their components, such as rRNA variants and stoichiometry of rRNA modifications, paralogs of ribosomal proteins, ribosome-associated proteins (for more detailed discussion, see (90)). These new layers of ribosome variability prompted the concept of specialized ribosomes and defects at one or more of their components are involved in several pathological conditions including cancer. Indeed, alterations in oncogenic or tumor suppressor factors may affect ribosome function to fine tune the translation machinery for its advantage, acquiring new potentialities in proliferation and progression. This gave rise to the concept of cancer specialized ribosomes or oncoribosomes (91). Alterations in rRNA modification together with alterations of snoRNAs expression, triggering tumor development and progression, could be the targets of novel therapeutic agents acting on these specialized oncoribosomes (92).

Altogether, the studies mentioned in this section indicate that altered snoRNAs expression in cancer can lead to changes in rRNA modification able to contribute to the neoplastic phenotype mainly affecting gene expression control at the translational level.

### Other alterations in snoRNA-mediated guide functions in cancer

In recent years, an increasing amount of experimental evidence has revealed that snoRNAs may have a functional role that extends far beyond their activity in ribosomal biogenesis. Specifically, these functions have been found to be involved in cancer development and progression. As mentioned, in addition to rRNA, snoRNAs can guide the modification complexes on different substrates including snRNAs, tRNAs and mRNAs. This last characteristic, i.e. the ability of snoRNAs to act as a guide for the modification of mRNAs, is suggested by the high number of transcripts found modified by dyskerin or fibrillarin (82,93,94). However, the experimental evidence associating the modification of residues to specific snoRNAs is still very limited. In one study, snoRNA-mediated pseudouridylation of mRNAs was observed at low levels, mainly due to the limited expression level of snoRNAs in the nucleoplasm and the length of the complementarity sequence between snoRNA and the target mRNA (95). In fact, a very limited number of mRNAs bearing an extended complementarity with snoRNAs was recognized so far. It was also investigated the effect of pseudouridine modification on premature termination codon, since in yeasts a remarkable readthrough was observed. By contrast, in human cells the readthrough was almost absent but further studies are required to explore the effect of pseudouridylation on mRNAs, which is now of great interest. Indeed, more recently another study revealed that H/ACA RNP complexes can associate with any newly transcribed mRNA, both endogenous and exogenous and that dyskerin can pseudouridylate target mRNA co-transcriptionally, independently of a guide RNA with perfect complementarity (94). Since H/ACA RNP complexes may enact a low level of pseudouridylation on target RNA also in presence of a certain degree of mismatch in the complementarity sequence, it has been proposed that mRNA pseudouridylation likely occurs with the assistance of other guide RNAs. Thanks to the binding to several different intronic Alu and long interspersed nuclear elements (LINEs) repeat stretches, it was suggested that dyskerin and GAR1 possibly assist in mRNA pseudouridylation. These RNA sequences in fact are highly structured as H/ACA box and can form stable AluACA complexes with H/ACA proteins (96). The large sequence variability of this presumed pseudouridylation loop of AluACA RNAs suggests a wide spectrum of target mRNAs, opening new potentialities for epitranscriptomic modifications. H/ACA complex-mediated pseudouridylation of mRNA may have direct functional effects on mRNAs translation, rather than its stability. Previous studies, in fact, demonstrated that pseudouridine modification in mRNA reduces translation rate as pseudouridine hinders tRNA correct accommodation in the ribosome (97) and induces premature termination due to ribosomal stalling (98). Anyway, the authors suggest that dyskerin-mediated pseudouridylation depends on the extent and the context of pseudouridine within the mRNA. Other recent studies have also demonstrated the 2′-O-methylation of mRNAs by the C/D box complex (99) with both SNORD32A (also known as U32A) and SNORD51 (U51) modifying the mRNA for PXDN, a peroxidase involved in oxidative stress. 2′-*O*-Methylation of *Pxdn* mRNA decreased the peroxidase activity in living cells by inhibiting translation. This observation strongly supports the role of posttranscriptional modification carried out by SNORD and fibrillarin for the regulatory control of gene expression. Furthermore, the stability of PARP-1 mRNA in endometrial cancer cells is significantly modulated by the expression of SNORD104, which is able to induce 2′-*O*-methylation in specific residues of PARP-1 mRNA thanks to its interaction with FBL (100). In fibroblast cell lines, SNORD113-6 was identified as the guide of the 2′-*O*-methylation of six integrin pathway mRNA by fibrillarin, which are essential for fibroblast interaction with other fibroblasts and with the extracellular matrix (101) and ultimately could be relevant for cancer progression (102,103).

The impact of modifications on U2 snRNA, such as 2′-*O*-methylation or pseudouridylation, is known to influence splicing efficiency. This occurs through the remodulation of branch point sequences, ultimately leading to alterations in gene expression (104). SCARNA15 can lead to the modification of Ψ-39 on the U2 of the spliceosome, allowing for alternative splicing events that are associated with cancer through the modification of some central pathways relating to tumor suppressors (such as ATRX and p53) as an adaptive response to stress (105).

tRNAs are the most modified class of RNAs (106), but only recently have some modifications been associated with the direct interaction of snoRNAs. Indeed, the cooperation between SNORD97 and SCARNA97 forms a C/D RNP complex that drives the 2′-*O*-methylation of the C34 wobble cytidine of human Met-tRNA (CAT) protecting against angiogenin-induced cleavage under stress (40). The previously mentioned SNORD113-6 not only drives the modification of certain mRNAs but also induces the 2′-O methylation of Leu-tRNA (TAA) (107), protecting it from its fragmentation and, consequently, to the production of tRNA-derived fragments (tRFs), which may play a role in the development of cancer (108). This mechanism was also found in another study where SNORD97 and SNORD133 drive 2′-*O*-methylation in multiple Met-tRNAs, inhibiting tFRs formation, altering the codon adaptation and gene expression related to proliferation and cellular development (109). These discoveries were achieved by using PARIS2, a highly optimized method for *in vivo* RNA duplex detection (110), unveiling numerous new snoRNA targets, both H/ACA box and C/D box within various ncRNAs types, including rRNAs, snRNAs, snoRNAs and nuclear-encoded tRNAs. It is therefore clear that evidence is accumulating on the role of snoRNAs in regulating tRNAs, particularly through 2′-*O*-methylation. In sum, these findings suggest that snoRNA plays a role in cancer not only by influencing modifications in rRNA but also exerting its impact on different RNA species, potentially affecting diverse processes such as mRNA translation and splicing.

### snoRNA interaction with cancer-related proteins

In addition to their guide functions on ribosomal and non-ribosomal rRNAs, snoRNAs have been implicated in cancer also through the direct interaction with protein complexes. Several years ago, a study demonstrated how SNORD50A and SNORD50B are directly linked to the K-Ras oncoproteins and their isoforms, suppressing their activity (111). In fact, the loss of these snoRNAs increases the association of K-Ras with GTPases leading to the activation of the ERK1/2 MAPK pathway. This dysfunction appears to be even more relevant as in numerous (10–40%) tumors a deletion in the gene locus of the SNORD50A and SNORD50B is frequently observed while their expression correlates with survival in patients with breast adenocarcinoma (111). A more recent study has also demonstrated how SNORD50A and SNORD50B directly bind to the protein complex of TRIM21 and GMPS (112), which are known to promote tumor progression by destabilizing p53 protein (113,114). Furthermore, SNORD50A shows a regulatory activity of 3′ mRNA processing. In particular, it can compete with the binding to Fip1 (a component of cleavage and polyadenylation specificity factor) thus resulting in a negative regulation of the targets of poly(A) processing at the 3′ end of mRNAs (48). These findings help to better understand the mechanism at the basis of SNORD50A involvement in cancer development (111,115–118). More recently it was found that SNORD17 promotes the growth and tumorigenicity of hepatocellular carcinoma cells (HCC) both *in vitro* and *in vivo* by modulating p53-mediated apoptosis (119). This effect is caused by a direct interaction within the nucleolus between SNORD17 and two new interactors: the nucleophosmin 1 (NPM1) and MYB binding protein 1a (MYBBP1A)—two proteins known to be involved in the activation of p53 (120,121). Notably, the loss of SNORD17 triggers the nuclear translocation of these proteins, thereby stabilizing the NPM1/MDM2 complex and initiating p53 activation mediated by MYBBP1A/p300. These findings suggest that SNORD17 drives cancer progression through the constitutive inhibition of p53 signaling. Another H/ACA box snoRNA (SNORA38B) has been proposed as an oncogene as it facilitates cell proliferation, migration, invasion and inhibition of apoptosis. SNORA38B plays a role in promoting tumor progression in NSCLC by directly binding to E2F transcription factor (E2F1) and regulating the GAB2/AKT/mTOR pathway thereby contributing to the development of an immunosuppressive tumor microenvironment. This occurs through its interaction with E2F1 in the nucleus, which controls the transcription of GAB2, subsequently activating the AKT/mTOR cascade (122). These articles emphasize that snoRNAs do not bind only to the canonical proteins of their respective RNP complexes, they also interact with various other proteins. For instance, PARP-1, a nuclear enzyme known to play a role in DNA repair and gene regulation (123), exhibits a preference for binding numerous H/ACA box snoRNAs in breast cancer cells (124). This binding enhances PARP-1 catalytic activity, promoting cell proliferation. Moreover, the orphan snoRNA SNORA73 can combine with PARP-1 and the H/ACA box RNP core proteins DKC1/NHP2 to form a snoRNP at DNA damage genomic *loci*, which blocks PARP-1 autoPARylation and DNA damage repair and leads to genome instability and cell differentiation in acute myeloid leukemia (51). Overall, this set of findings clearly indicate that snoRNA can interact through different mechanisms with several cellular players involved in cancer also independently of their effect on RNA modification.

### snoRNA retaining transcripts as unconsidered players in cancer

In addition to what has been reported so far, a previously overlooked role for snoRNAs is emerging. In fact, they can also be detected incorporated into longer and more heterogeneous RNA molecules with unconsidered physiological roles. Generally, during the snoRNA biogenesis, circumstances may arise where the snoRNA undergoes non-canonical maturation and can be retained alongside various portions of the host gene transcript. In recent years, more and more different types of these transcripts are being discovered, each termed in different ways, which we can generally name as snoRNA retaining transcripts or snoRTs. The first type to be identified was defined as sno-lncRNA by Ling-Ling Chen's group. These nuclear RNAs are generated through exonucleolytic trimming of the host gene, resulting in an intron containing two snoRNAs (either C/D box or H/ACA box) at the two ends of the transcript. Consequently, sno-lncRNAs lack a 5′ m7G cap and a poly(A) tail (125). This category was subsequently enriched by a specific transcript containing SNORA5A and SNORA5C sequences at its ends which directly impacts rRNA transcription and for this reason defined as SLERT (snoRNA-ended lncRNA enhances pre-ribosomal RNA transcription) (126). In fact, this transcript acts as a chaperone of the DEAD-box helicase DDX21 (127) thanks to a non-snoRNA sequence of 143 nucleotides inducing the transcription of the rDNA by the RNA polymerase (Pol) I. This occurs because the clusters of DDX21 that coat the dense fibrillar component in the nucleoli are loosed by the presence of SLERTs, a necessary step for the liquidity of the border between the fibrillar centers and the dense fibrillar component, enhancing the processivity of RNA Pol I (127). Thus, deletion of a SLERT impairs pre-mRNA transcription and rRNA production, resulting in decreased tumorigenesis (126). The same research team has also identified a group of snoRTs that are capped by snoRNA at their 5′ end (either C/D box or H/ACA box) and are 3′ poly-adenylated, termed for this reason as SPAs (snoRNA capped and 3′ polyadenylated) (128). These transcripts are formed through a competitive and co-transcriptional process between the degradation by XRN2 and the elongation due to RNA Pol II of the pre-mRNA from which they derive. The presence of the snoRNA sequence with the rest of the RNPs ensures the termination of the degradation and the accumulation in the nucleus. SPAs bind to other RNA binding proteins, ultimately resulting in an alteration in the alternative splicing pattern associated with Prader–Willi syndrome (128). These SPA transcripts were further studied in cytoplasmic variants with potential implications in ribosomal biogenesis and cancer development. In fact, a particular transcript of NOP56 retaining snoRD86 sequence allows the fine tuning of the production of NOP56 through alternative splice donors in its pre-mRNA (129). In this context, the presence of NOP56 alongside other RNPs on the snoRD86 sequence of the pre-mRNA, plays a pivotal role in the formation of the transcript with the retained intron. This prevents the formation of the coding RNA and therefore regulating the expression of NOP56 itself. Eventually, this transcript is free to go into the cytoplasm and be further degraded thanks to NMD (14) and therefore become a cytoplasmic SPA (cSPA). This cytoplasmic lncRNA class was further enriched by H/ACA box snoRNA snoRTs (130). A high number of these transcripts bind dyskerin in the cytoplasm, some of them in the form of SPA RNA in high abundance. These transcripts are suggested to play a potential role in cytoplasmic RNA regulation by exerting dyskerin functions beyond its known role in the nucleus. In particular, they could act as a guide for the post-transcriptional modification of some mRNAs by the pseudouridylation RNP complex in the cytoplasm, regulating the translation of key mRNAs for the development of breast cancer, including some involved in nuclear hormone receptors. For these reasons the lack of cytoplasmic dyskerin may represent a way to escape hormone dependence in breast cancer cells and in patients with estrogen positive status (130). Remaining in the cytoplasmic context, a further typology of snoRTs has been reported, characterized by the formation of lariats bearing a snoRNA, thus termed as slb-snoRNAs (131). These transcripts are associated with RNPs but do not guide post-transcriptional modification, but rather are partly exported, thus engaging in a competitive action with snoRNA maturation.

The presence of many cytoplasmic snoRTs, some of which have mRNAs characteristics (such as the presence of a poly(A) tail), suggests that their significance may have been frequently overlooked by high-throughput sequencing systems, ultimately classifying them as canonical snoRNAs. Regarding the possible structural variants of snoRTs, there is the instance of a long non-coding RNA (lncRNA) named LNC-SNO49AB, with SNORD49A and SNORD49B sequences, a 5′ m7G cap, and a snoRNA at its 3′ terminus (132). Its interaction with fibrillarin in the nucleolus is not associated with the 2′-*O*-methylation activity, but rather with the stability of the transcript itself. Remarkably, LNC-SNO49AB facilitates the post-translational dimerization of adenosine deaminase acting on RNA 1 (ADAR1) in the nucleolus. This stimulation enhances the global A-to-I RNA editing rate, influencing epitranscriptomic alterations in cancer. Notably, the expression of LNC-SNO49AB correlates with leukemia progression both *in vitro* and *in vivo*.

**Figure 3.**
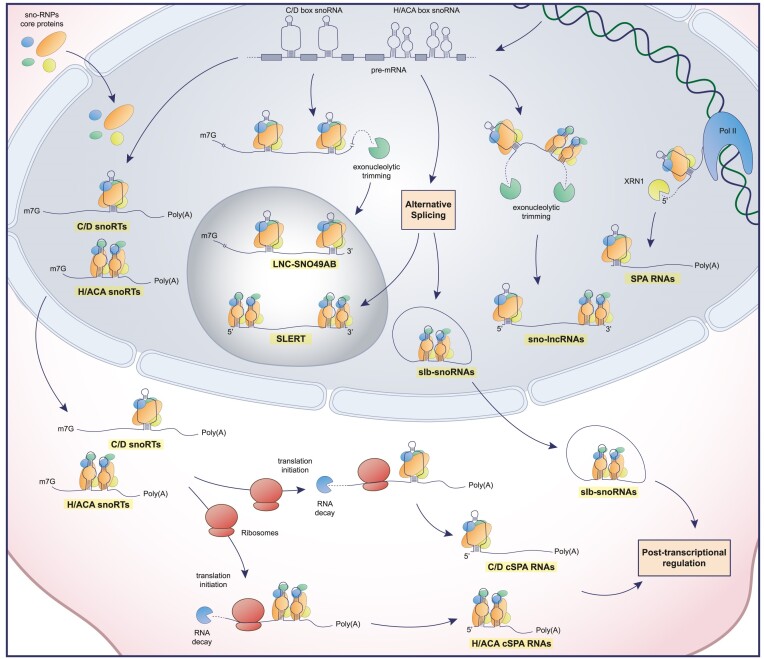
Schematic representation of snoRTs biogenesis. snoRTs are processed from pre-mRNA or co-transcriptionally derived (SPA RNAs) and are subsequently processed and differentially localized in the cell. Poly(A): poly-adenine tail; m7G: mRNA cap; Pol II: RNA polymerase II; XRN1: 5′-3′ exoribonuclease 1.

In sum, this last set of studies on one side identifies a number of previously unconsidered functions of snoRNAs when they are retained in longer transcripts and, on the other side, clearly point out that a considerable amount of snoRNA sequences is present in cells and tissues in non-canonical forms. Figure 3 and Table 1 summarize the mentioned features of snoRTs and their potential implications in cancer and other disorders. On these bases, caution should be exerted when considering snoRNA value as diagnostic/prognostic biomarkers since the most common approaches for their quantification may not be able to distinguish among the various forms in which they could be represented.

**Table 1.** Types of described snoRNA retaining transcripts (snoRTs)

Name	snoRNA box	Localization	Size (Kb)	Poly (A)	Involvement in disease	References
sno-lncRNA	C/D - H/ACA	Nucleus	12 - 22	No	Prader-Willi Syndrome	(125)
SLERT	H/ACA	Nucleolus	0.7	No	Development of neoplastic features	(126,127)
SPA RNA	C/D	Nucleus	16–34	Yes	Prader-Willi Syndrome	(128)
C/D snoRT - cSPA	C/D	Cytoplasm	1.3–1.8	Yes	Aberrant ribosomal biogenesis	(129)
H/ACA snoRT - cSPA	H/ACA	Cytoplasm	0.3–10	Yes	Estrogen independent tumor progression	(130)
slb-snoRNA	C/D–H/ACA	Cytoplasm	0.3–1.2	No	Regulation of snoRNAs expression in cancer cells	(131)
LNC-SNO49AB	C/D	Nucleolus	0.8	No	Leukemia progression	(132)

## Conclusion and perspectives

In conclusion, the data available provide a significant body of information to understand the role of snoRNAs in the physiological context. Concerning their role in cancer, in literature a constantly growing amount of studies provide limited insight (and in fact a huge background noise effect) since they have been conducted without considering all the technical issues related to snoRNA quantification and without attempting to provide functional validations of the results obtained by the analysis of patient derived material. Moreover, the quantification of snoRNAs may not always have been reliable considering that they are frequently present as retained sequences in larger transcripts. However, focusing on technically and functionally validated studies it is possible, already at this stage, to conclude that dysregulation in snoRNA expression and metabolism can contribute at many levels to the cancerous phenotype, as it is reported in the different sections of the present review. This general consideration holds an extreme potential since, having each snoRNA unique sequence features, they in principle represent viable targets for therapeutical purposes to different ends, including the selective decrease of their expression, the impairment of their guide function and/or the modification of the steps required for their biogenesis. Recently, several clinical trials targeting mRNAs and miRNAs have been approved (133). These studies use approaches that could also be exploited to target snoRNAs, such as e.g. siRNAs and LNA antisense oligonucleotides (ASO). They could be designed to recognize specifically the unique sequence of each target snoRNA or their direct interactors. As a proof of concepts, there are already promising preclinical studies in mice models that exploit these techniques to target snoRNAs relevant in colon and lung cancer (134,122). Considering that we are approaching an era in which more and more strategies will become available to specifically target RNA molecules (also on the basis of their cellular interactors and localization), it appears necessary to further characterize the role of snoRNAs in cancer.

## Data Availability

No new data were generated or analysed in support of this research.
